# Classification of fundus autofluorescence images based on macular function in retinitis pigmentosa using convolutional neural networks

**DOI:** 10.1007/s10384-025-01163-w

**Published:** 2025-02-12

**Authors:** Taro Kominami, Shinji Ueno, Junya Ota, Taiga Inooka, Masahiro Oda, Kensaku Mori, Koji M Nishiguchi

**Affiliations:** 1 Department of Ophthalmology, Nagoya University Graduate School of Medicine, 65 Tsuruma- cho, Showa-ku, Nagoya, 466-8550 Japan; 2 Department of Ophthalmology, Hirosaki University Graduate School of Medicine, Hirosaki, Japan; 3 Graduate School of Informatics, Nagoya University, Nagoya, Japan; 4 Information Technology Center, Nagoya University, Nagoya, Japan; 5 Research Center for Medical Bigdata, National Institute of Informatics, Nagoya, Japan

**Keywords:** Fundus autofluorescence image, Retinitis pigmentosa, Convolutional neural networks, Machine learning, Central visual function

## Abstract

**Purpose:**

To determine whether convolutional neural networks (CNN) can classify the severity of central vision loss using fundus autofluorescence (FAF) images and color fundus images of retinitis pigmentosa (RP), and to evaluate the utility of those images for severity classification.

**Study design:**

Retrospective observational study.

**Methods:**

Medical charts of patients with RP who visited Nagoya University Hospital were reviewed. Eyes with atypical RP or previous surgery were excluded. The mild group was comprised of patients with a mean deviation value of > − 10 decibels, and the severe group of < − 20 decibels, in the Humphrey field analyzer 10-2 program. CNN models were created by transfer learning of VGG16 pretrained with ImageNet to classify patients as either mild or severe, using FAF images or color fundus images.

**Results:**

Overall, 165 patients were included in this study; 80 patients were classified into the severe and 85 into the mild group. The test data comprised 40 patients in each group, and the images of the remaining patients were used as training data, with data augmentation by rotation and flipping. The highest accuracies of the CNN models when using color fundus and FAF images were 63.75% and 87.50%, respectively.

**Conclusion:**

Using FAF images may enable the accurate assessment of central vision function in RP. FAF images may include more parameters than color fundus images that can evaluate central visual function.

## Introduction

Retinitis pigmentosa (RP) is a type of inherited retinal dystrophy (IRD) that causes peripheral visual field loss due to the degeneration of rod photoreceptors as well as severe visual acuity impairments due to the degeneration of cone photoreceptors [1]. Worldwide, RP has a frequency of approximately 1:4,000 persons [2, 3]. RP is an intractable disease; photoreceptor transplantation such as retinal prosthesis [4, 5], regenerative medicine using induced pluripotent stem cells or embryonic stem cells [6, 7], gene therapy [8–11], neuroprotection [12], and optogenetics [13] are potential treatments for RP depending on the disease stage; however, it is difficult to evaluate their effectiveness. One reason is that clinical trials are conducted for a limited number of years. As the progression of RP is slow, some of these clinical trials fail to demonstrate the effectiveness of treatment due to the short study period. Additionally, identifying parameters of visual function is important for evaluation leading to the development of new treatments. Imaging technology, such as fundus photography and optical coherence tomography (OCT), are objective indicators of visual function that are noninvasive and less burdensome on patients. We hypothesized that with a parameter that reflects visual function in the fundus image, it would be possible to classify visual function by fundus image. Artificial intelligence (AI), which includes machine learning techniques such as deep learning and its subset convolutional neural networks (CNN), is highly compatible with image analysis and has enabled advances and innovations with hardware such as graphic processing units (GPU). AI has been employed in studies on RP or IRD [14–18] as well as other eye diseases such as glaucoma [19, 20]. Liu et al. utilized transfer learning to predict visual acuity based on fundus images [16], while Nagasato et al. evaluated a deep learning model that predicted visual function parameters, including visual field tests from fundus image [18]. However, a CNN model to classify the severity of central visual function using fundus images of RP has not been completely analyzed. Therefore, the primary purpose of this study was to evaluate the accuracy of CNN models in the classification of fundus images based on macular function. Specifically, the study also aimed to determine the utility of FAF and color fundus images to assess visual function, which could potentially improve diagnostic accuracy and patient management in clinical settings. To accomplish this, we created CNN models to classify fundus autofluorescence (FAF) images and color fundus images labeled as either severe or mild determined by perimetry. We demonstrated the utility of FAF imaging in classifying the severity of macular function in RP.

## Patients and methods

### Patient selection

This retrospective observational study was conducted at Nagoya University Hospital. All procedures conformed to the tenets of the Declaration of Helsinki and were approved by the Institutional Review Board/Ethics Committee of Nagoya University Hospital (approval number: 2023-0382). This study allowed patients to refuse participation by opt-out instead of obtaining written informed consent.

RP was diagnosed based on the ocular history, concentric contraction of the visual field, pathognomonic features of the fundus (e.g., pigmentary changes and attenuation of blood vessels), and a reduced response of electroretinograms.

In this study, medical records of 326 patients with RP who visited Nagoya University Hospital between February 2007 and March 2023 were reviewed. Of these patients, those who underwent fundus color photography and FAF imaging with an ultra-wide field imaging device (Optos P200Tx; Optos) and Humphrey Field Analyzer (HFA; Carl Zeiss Meditec, Inc.) test 10-2 program were enrolled.

We excluded eyes with atypical RP, such as sectorial RP or nonpigmented RP, vitrectomized eyes, and eyes with macular holes.

None of the included eyes had severe epiretinal membranes. Patients were divided into two groups, severe and mild, according to the criteria described below. We also evaluated whether there were any differences between these two groups in terms of sex ratio, age at the last visit, phakia to pseudophakia ratio, and the ratio of eyes used in the analysis [both eyes (OU), right eye (OD), or left eye (OS)].

### Severity classification

Based on previous reports showing that HFA 10-2 program was useful in evaluating visual function in patients with RP [21–23], we defined images with a mean deviation (MD) value of > − 10 and < − 20 decibels (dB) on the HFA 10-2 program as the mild and severe groups, respectively. Whenever an HFA 10-2 test was performed within a year of the image acquisition date, that MD value was used to determine the classification of the image. Meanwhile, if no HFA 10-2 test had been performed within a year, the classification was performed only when both the MD values of before and after the image acquisition date were determined to be the same, either mild or severe value.

OU images were included only if they were classified into the same group, whereas OD images were included only if the OD and OS images were classified into different groups.

Images taken on different dates that met the criteria for HFA 10-2 were included even if they were from the same person and eye; however, with patients who had undergone cataract surgery, only images taken after the surgery were included.

There was one case that had initially been determined to be mild but was later reclassified as severe; in this case, only images determined to be severe were analyzed.

OD images were included in the analysis only when the OD and OS had different lens conditions (e.g., phakia in OD and pseudophakia in OS).

### Dataset

In both groups, 40 cases were randomly selected, and only one image of each case was randomly extracted as test data. The image was cropped to retain the central half, which was vital for assessment of central vision function. This approach should enhance the accuracy of the model by concentrating on the most relevant parts of the image for assessing macular function. The extracted images were used as test data to confirm the performance of the CNN model. Images of the remaining cases were used as training data. The training images were cropped so that the center half remained similar to the test images and were augmented 16 times by rotation and flipping.

### Deep learning model

We created a CNN model to classify the fundus color or FAF images into the severe and mild groups. CNN models for FAF images and fundus color images were created separately. This study used visual geometry group-16 (VGG16) to create the CNN [24]. All images were resized to 228 × 228 pixels. Transfer learning was applied to VGG16 using the initial weights obtained from training on the ImageNet dataset. The last output layer was replaced with several patterns, all with a final dense layer for binary classification. Optimization was performed using the Adam optimizer with several learning rate patterns. The loss function was categorical cross-entropy. The models were trained for up to 1,000 epochs with 32 images per step. We examined fivefold cross-validation of the training data to create an optimal CNN model. The model created for the training images was used to determine whether the test image was severe or mild (the group for which the probability was determined to be ≥ 0.5 was used as the prediction result). Under the same conditions, transfer learning using Xception [25], DenseNet201 [26], and MobileNet [27] were preliminarily evaluated to determine whether the CNN models outperformed VGG16 for this present study.

All models were trained on a computer with Ubuntu (18.04), Intel^®^ Xeno Gold 6134 Computer Processing Unit, four Quadro P6000 GPUs, and 192 GB system memory. The CNN models were developed with Python Keras (https://keras.io/ja/) using TensorFlow (https://www.tensorflow.org/) as the backend.

### Heatmaps

To visualize where the CNN model was focused, heat maps were created and were overlaid on the corresponding fundus images. Gradient-weighted Class Activation Mapping (Grad-CAM) was used [28]. The target layer was the third convolutional layer in block 5.

### Metrics

To evaluate the model performance, we calculated the accuracy and area under the receiver operating characteristic curve (AUC) with 95% confidence interval (CI) as described by the true positive rate (sensitivity)–false positive rate (1-specificity). Accuracy is the ratio of the number of correctly predicted images to the total number of images. To estimate the 95% CI for the AUC, we performed bootstrap resampling with 1,000 iterations.

### Statistical analysis

The chi-square test was employed to determine the differences in the male–female, phakia–IOL, and OU–OD–OS ratios between the severe and mild groups. The Mann–Whitney U test was used to compare the age at the last visit in the severe and mild groups. Python Scipy (https://scipy.org/) and Python Statsmodels (https://www.statsmodels.org/stable/index.html) were used for statistical analyses.

## Results

**Fig. 1:**
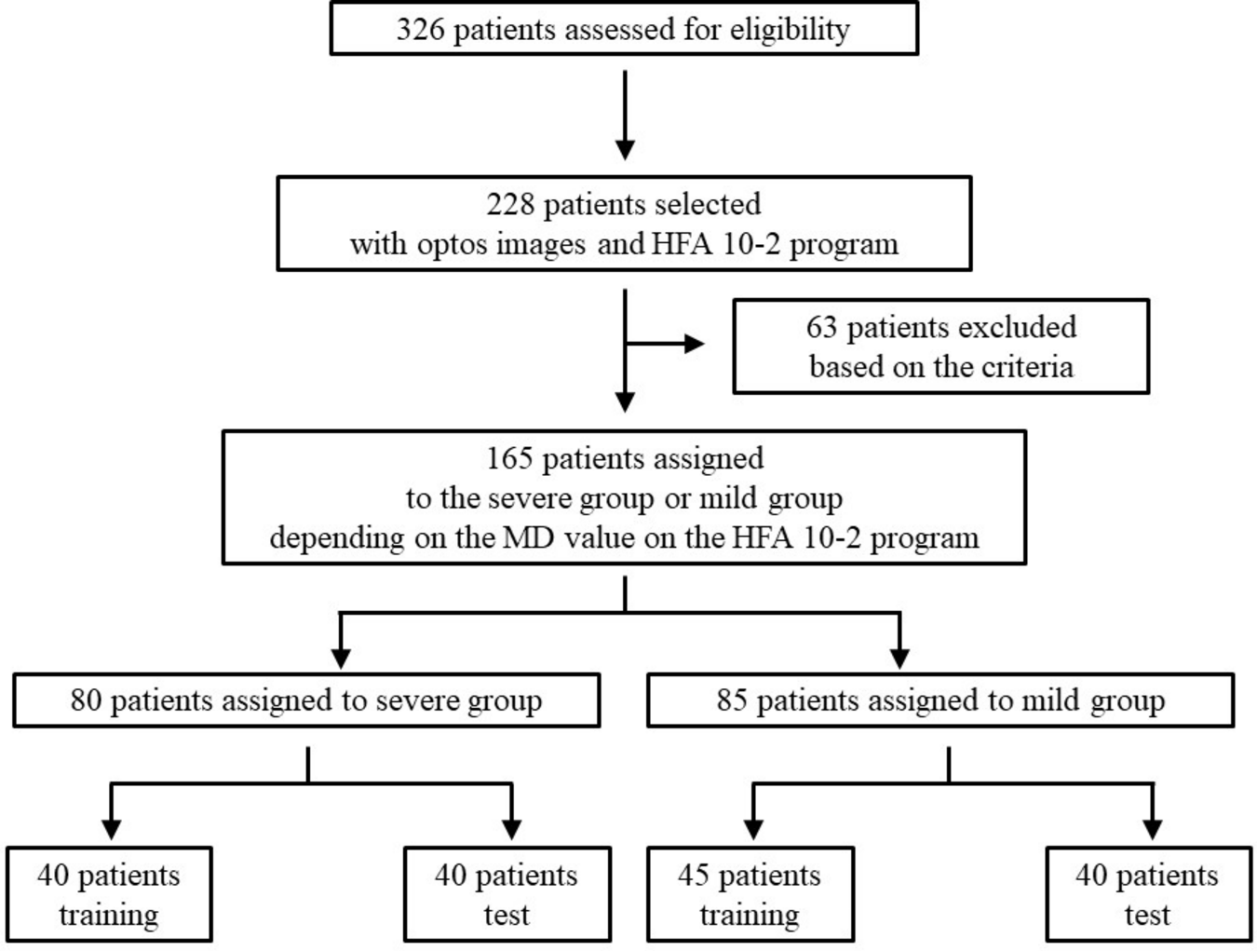
Flowchart of patient selection and assignment.*HFA* Humphrey field analyzer, *MD* mean deviation

The medical records of 326 patients with RP were reviewed. As shown in Fig. 1, we identified 228 patients who underwent both fundus imaging and HFA 10-2. After applying the exclusion criteria, 165 patients (74 men, 91 women) were finally included in the analysis. The demographic characteristics of the patients are presented in Table 1. Overall, 80 patients comprised the severe and 85 the mild group. There were no statistically significant differences between both groups regarding sex ratio, age at the last visit, phakia to pseudophakia ratio, or the ratio of eyes used in the analysis (OU, OD, or OS). As 40 cases in the severe and mild groups were used for testing, 40 in the severe and 45 in the mild” group comprised the training data for the CNN models. In total, 324 original FAF and 357 original color fundus images were collected before data augmentation for training. After applying augmentation techniques (rotation and flipping), the final training dataset comprised 5184 FAF images and 5712 color fundus images.

**Table 1:** Demographic characteristics of the patients

	Severe	Mild	*p*-value
Total number	80	85	NA
Sex (male/female)	36/44	38/47	0.91
Age at last visit (ave ± SD)	54.09 ± 15.54	53.53 ± 18.75	0.40
Lens (phakia/IOL)	52/28	63/22	0.27
Analyzed eye (OU/OD/OS)	65/9/6	62/16/7	0.38

*Ave* average, *SD* standard deviation, *IOL* intraocular lens, *OU* both eyes, *OD* right eye, *OS* left eye

### CNN model performance

The representative parameters of epochs, learning rates, dense layers, and accuracy of the CNN models for FAF image classification are listed in Table 2. Based on the experiments, approximately 50 epochs were sufficient to improve the accuracy of the CNN model in predicting the severity of FAF images. The learning rate of 0.00001 was considered better to predict the severity of RP using FAF images. Accuracy improved to 76.25% even at 3 epochs compared with the learning rate of 0.0001. For the dense layer, the accuracy was superior at 87.50% with a single-layer structure of 1024 units. For color fundus images, accuracy was up to 63.75% for any parameter.

**Table 2:** Representative parameters of the CNN models for FAF image classification

Epochs	lr	Dense layers	Accuracy (%)
3	0.0001	2048→512→256	50
3	0.00001	2048→512→256	76.25
25	0.00001	2048→1024→512→256	75
25	0.00001	1024→512→256	81.25
25	0.00001	512→256	77.5
25	0.00001	512	82.5
25	0.00001	1024	87.5
25	0.00001	2048	85
200	0.00001	1024	85
1000	0.00001	1024	85

*CNN* convolutional neural network, *FAF* fundus fluorescence image, *epochs* used for training, *lr* learning rate, *Accuracy* accuracy of test data

**Fig. 2:**
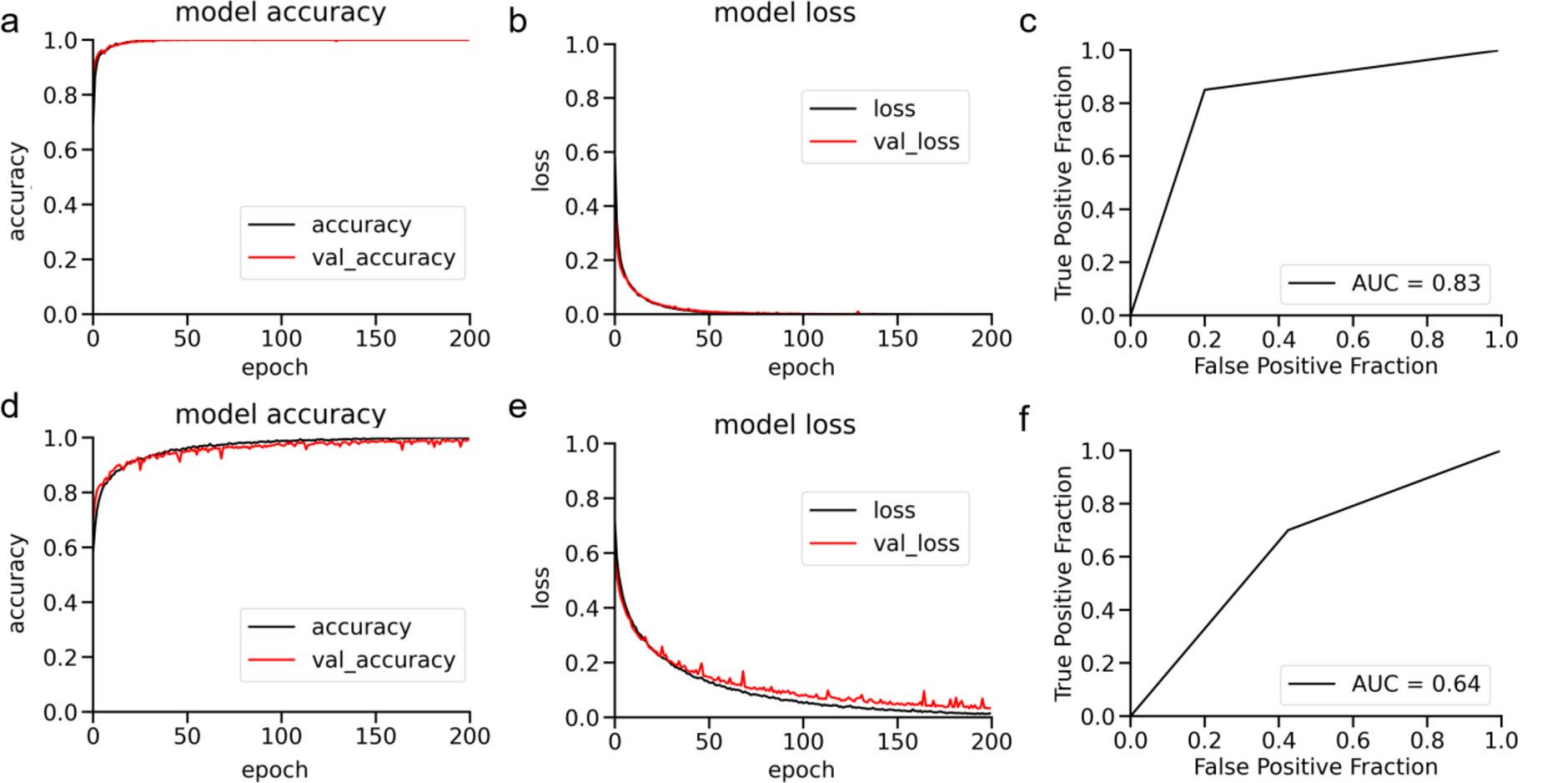
Transitions of accuracies for the first dataset in fivefold cross-validation in fundus autofluorescence (FAF) images (**a**) and color fundus images (**d**), those of the loss function in FAF images (**b**) and color fundus images (**e**), and receiver operating characteristic curves in FAF images (**c**), and color fundus images (**f**). The black and red lines in 4a–4e represent the results of the training and validation datasets, respectively. *AUC* area under the curve, *val* validation

The representative transitions of accuracy and loss of function for the first dataset in 5-fold cross-validation and the receiver operating characteristic curve of the test data in FAF and color fundus images with a learning rate of 0.00001 and a single dense layer of 1,024 units are shown in Fig. 2.

In FAF image training, accuracy (Fig. 2a) and loss (Fig. 2b) reached a plateau in approximately 50 epochs. The representative AUC for FAF image prediction was 0.83 (95% CI [0.741, 0.900]) (Fig. 2c). In contrast, when training for color fundus images, approximately 100 or 200 epochs were required for each accuracy (Fig. 2d) and loss (Fig. 2e) to reach the plateau. The representative AUC for color fundus images was 0.64 (95% CI [0.524, 0.737]) (Fig. 2f). The 95% CIs for the AUCs of FAF and color fundus images did not overlap.

For preliminary analysis, transfer learning was employed using Xception [25], DenseNet201 [26], and MobileNet [27] to obtain a CNN model with better accuracy; however, the highest accuracy was approximately 70% with DenseNet201 or MobileNet, while the accuracy using the Xception model was slightly lower than that of VGG16.

### Heatmaps

**Fig. 3:**
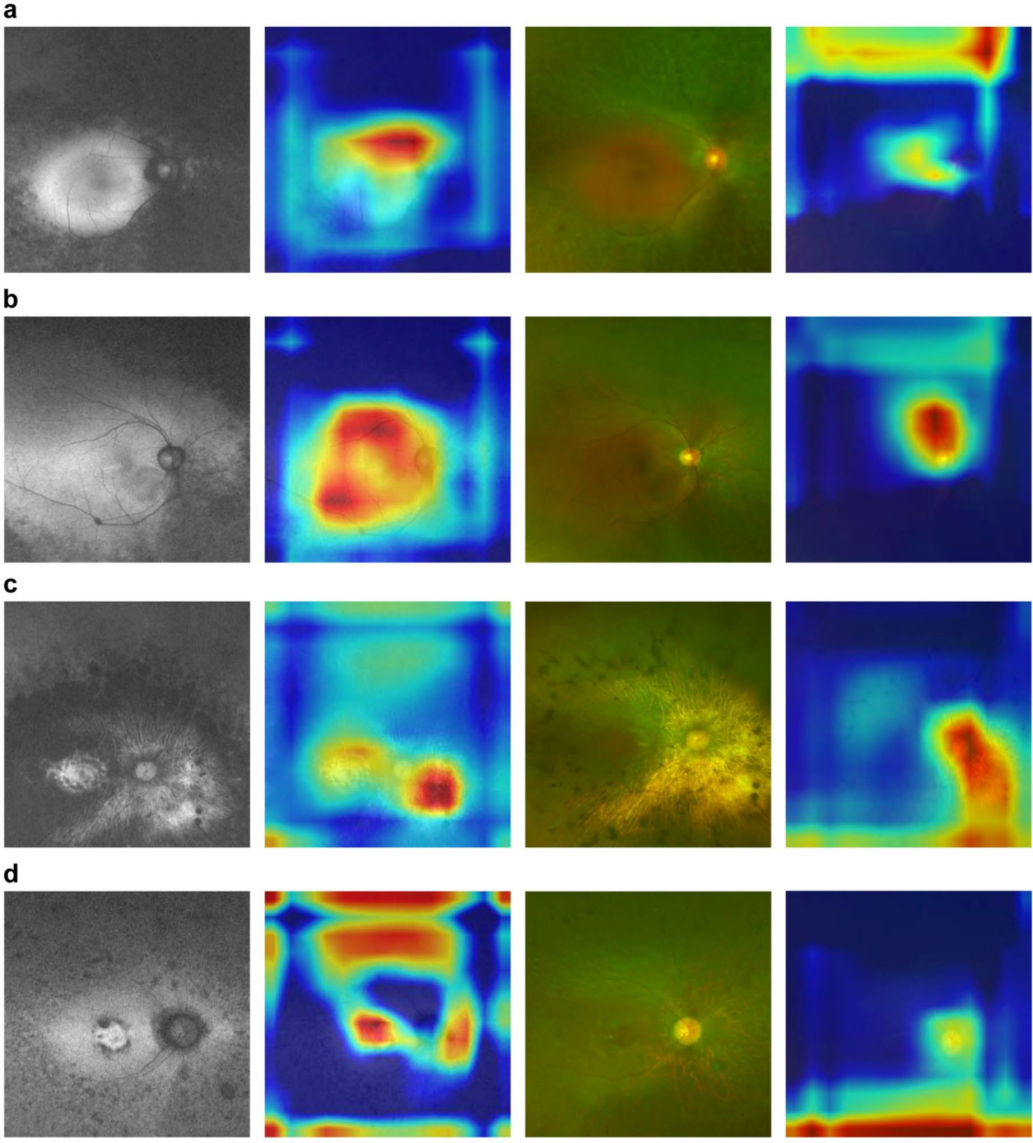
Representative correctly predicted fundus autofluorescence (FAF) images and color fundus photographs with heatmaps weighted by Gradient-weighted Class Activation Mapping (Grad-CAM). **a** shows images of the right eye (OD) of a 53-year-old woman whose mean deviation value (MD) of the Humphrey field analyzer (HFA) 10-2 program was better than − 6.58 decibels (dB), and **b** shows the OD images of a 52-year-old man whose MD of the HFA 10-2 program was − 0.25 dB. These images were labeled “mild” and were correctly predicted as “mild” by the deep learning (DL) model. **c** shows OD images of a 71-year-old man whose MD of the HFA 10-2 program was worse than− 24.66 dB, and **d** shows OD images of a 22-year-old woman whose MD of the HFA 10-2 program was worse than − 20.38 dB. These images were labeled as“severe” and were correctly predicted as “severe” by the DL model. Heatmaps are displayed next to each FAF image and the color fundus photograph. The warmer the colors, such as red, the stronger the weighting of the DL model, and the colder the colors such as blue, the weaker the weighting of the DL model

Figure 3 shows examples of correctly predicted FAF images and color fundus photographs with heatmaps weighted by Grad-CAM of the CNN model as displayed in Fig. 2. Figure 3a, b, c and d show the representative images of mild and severe cases, respectively. The CNN models revealed the macula in all the FAF images. In representative severe cases, the CNN model also focused on the area outside the arcade vessels as well as the macula (Fig. 3c and d). Conversely, in the color fundus images, CNN models revealed not the macula but the areas above and below the optic disk as well as more peripheral regions in both mild and severe cases.

**Fig. 4:**
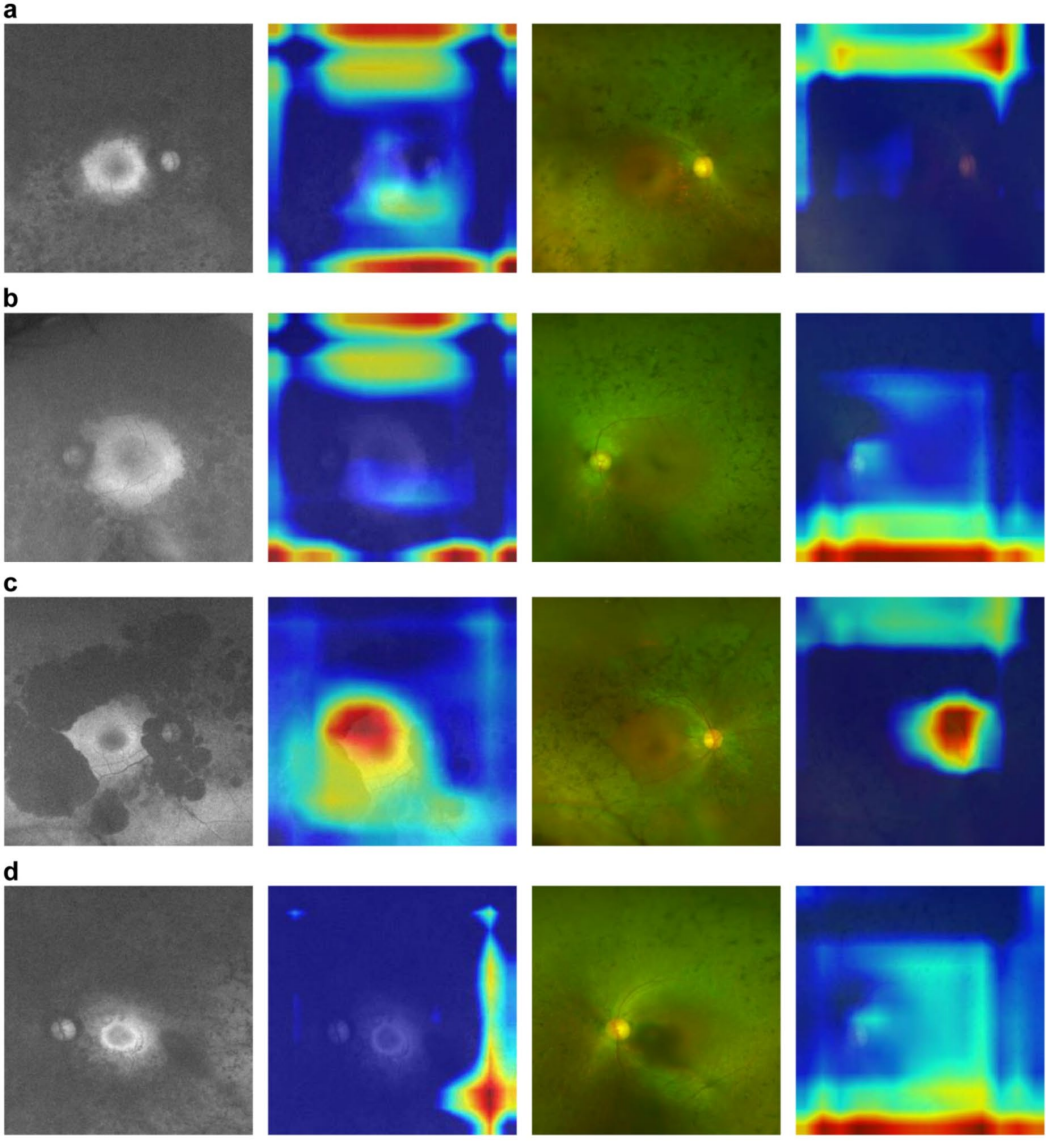
Representative incorrectly predicted fundus autofluorescence (FAF) images and color fundus photographs with heatmaps weighted by Gradient-weighted Class Activation Mapping (Grad-CAM). **a** shows images of the right eye (OD) of a 71-year-old man whose mean deviation value (MD) of the Humphrey field analyzer (HFA) 10-2 program was better than − 9.49 decibels (dB), and **b** shows images of the left eye (OS) of a 63-year-old woman whose MD of the HFA 10-2 program was − 7.50 dB. These images were labeled as “mild” but were incorrectly predicted as “severe” by the deep learning (DL) model. **c** shows OD images of a 73-year-old man whose MD of the HFA 10-2 program was − 22.21 dB. These images were labeled as “severe” but were incorrectly predicted as “mild” by the DL model. **d** shows OS images of a 50-year-old woman in whom MD of the HFA 10-2 program was − 21.97 dB. These images were labeled as “severe,” and the color fundus photograph was correctly predicted as “severe”; however, the FAF was incorrectly predicted as “mild” by the DL model. Heatmaps are displayed next to each FAF image and the color fundus photograph. The warmer the colors, such as red, the stronger the weighting of the DL model, and the colder the colors such as blue, the weaker the weighting of the DL model

Figure 4 shows examples of incorrectly predicted FAF images and color fundus photographs with heatmaps weighted by Grad-CAM of the CNN model made as shown in Fig. 2. Figure 4a, b, c and d show the representative images of mild and severe cases, respectively. Unlike the correctly predicted FAF images, CNN models did not take account of the macula in most cases of incorrectly predicted FAF images. FAF images labeled as mild but had been predicted as severe implied that the CNN model focused on the area outside the arcade vessels. Figure 4c shows the FAF image labeled as severe that had been predicted to be mild, demonstrating that the CNN model focused on the macula. In contrast, In the color fundus images, CNN models focused on the area above the optic disc or more peripheral areas, similar to the correctly predicted color fundus images. Unlike other images, the color fundus image in Fig. 4d was correctly predicted as severe.

## Discussion

This study demonstrated the potential of CNNs to classify FAF images based on the severity of RP on the HFA10-2 program. However, CNN models for color fundus images did not perform well. These results are consistent with those of a previous study in which FAF images showed good performance in estimating the visual function of the RP [18]. Additionally, FAF images are reportedly useful for classifying IRDs [15, 29]. FAF may also detect abnormalities beyond those revealed on funduscopic examination, fluorescein angiography, or OCT and are helpful for differential diagnosis, detection, and extent delineation of involved retinal areas, genotype–phenotype correlations, and monitoring of changes over time [30]. For example, the Robson–Holder ring, one of the major FAF phenotypes, can not be visualized on color fundus images [31]. FAF may be a better imaging method for detecting IRD progression than color fundus imaging.

The CNN model in this study exhibited an accuracy of 87.50% for FAF images, comparable to that of a previous study that used deep learning to predict visual acuity via confocal scanning laser ophthalmoscopy imaging [16]. Notably, the accuracy may vary depending on the resolution of the images or cropping. Uncropped 512 × 512-pixel images show hyper-performance in estimating the visual function of patients with RP [18]. Although the accuracy might be better with other CNN models, with other hyperparameter settings, or with multimodal learning combined with other factors such as visual acuity, OCT parameters, or other methods to crop or with other image resolutions, it is challenging to perform analyses with truly optimal hyperparameters because there is an infinite number of hyperparameter settings.

Although the intrinsic focus of the CNN model is unclear, Grad-CAM can partly show where the CNN model focuses on fundus imaging [28]. In color fundus imaging, it is difficult to determine where the CNN model focuses when classifying the severity of RP, whereas in FAF images the CNN model focuses on the macular region. It seems reasonable that the CNN models for FAF images focused on the macular region because in this study severity was determined based on the HFA 10-2 program MD value. Furthermore, the CNN models for FAF images might focus on the area outside the macula such as the arcade vessels. The CNN models mainly focused on the macula in FAF images of the mild cases; conversely, the CNN model seemed to focus on the macula as well as on the area outside it in the severe FAF images. Additionally, some FAF images were incorrectly predicted. As shown in Fig. 4a and b, FAF images were incorrectly predicted as severe despite being labeled as mild. In these images, the CNN models did not focus on the macula but on the area outside it, and it was difficult to detect the arcade vessels in these FAF images. In contrast, the FAF images correctly predicted as mild clearly showed the arcade vessels as shown in Fig. 3b. There might be a tendency to classify images as severe if characteristic changes were present outside the macula, such as the arcade vessels, and mild if there were little changes outside the macula. As RP is considered a centripetal progression of retinal degeneration to the macula, it is reasonable that arcade vessels are less likely to be detected in FAF images of more advanced RP, such as those with severe central macular function. The actual reason for the presence of incorrectly predicted images remains unclear, but in RP, wherein phenotypic differences are reported even for the same pathogenic gene such as *RP1* [32], there might be individual differences between progression of retinal degeneration of the function and structure, and the CNN model might not be able to correctly determine pronounced differences. Another possible explanation for the incorrect prediction of FAF images is that macular degeneration may accompany macular involvement in the early stages of RP, although RP is typically associated with afferent retinal degeneration [33]. Thus, some cases, such as those in Fig. 4c, may be labeled as severe due to poor central visual field function, even if they would be categorized as mild based on the visible arcade vessels. Creating a CNN model that can further accurately classify may be possible by increasing the number of cases. For color fundus images, there was no obvious difference between correctly and incorrectly predicted images which the CNN models recognized. This observation is consistent with the lower accuracy of CNN models for color fundus images than for FAF images. Unlike FAF images, color fundus images are less prone to feature changes that reflect central visual dysfunction; thus, heatmaps may also show only nonspecific changes other than those in the macula.

### Limitations

This study had several limitations. The number of cases analyzed in this study was limited because of the rarity of RP; thus, it may not be sufficient to create an excellent CNN model. In the future, a better CNN model may be created by accumulating more cases in a multicenter study. This study did not consider phenotypic changes due to the pathogenic genes of RP, which include more than 80 genes. This indicates that, in studies like ours where the pathogenic gene is not standardized, greater variability in phenotypes may occur, potentially lowering the classification accuracy of the CNN model. However, patients with the same pathogenic gene may not always exhibit similar phenotypes as described above. The images were cropped to exclude peripheral areas to enhance model performance by concentrating on the most diagnostically relevant region (macula) and reducing noise (such as eyelids, eyelashes, or fingers) from less relevant peripheral areas, thereby potentially increasing classification accuracy. However, exclusion of the peripheral retina might make it impossible to observe critical structural changes in the periphery and may impact the accuracy of the evaluation of fundus images because the CNN models in some of the fundus images in this study focused on areas outside the macula, such as the arcade vessels, as well as the macula. This study used ultra-wide field fundus imaging; however, conventional color fundus photographs might better reflect structural changes. Although FAF images sometimes become blurred as RP progresses, this study did not exclude FAF images of patients with progressive RP.

In conclusion, this study demonstrates the possibility of using FAF images in CNN models to classify the severity of RP depending on macular function, which might imply that FAF images are better tools for predicting macular function than color fundus imaging.

FAF may also play an essential role in algorithms for determining treatment efficacy in future clinical trials, which may lead to medical innovations in the development of future treatments for RP. For this, it might be desirable to perform further analysis with an improved method, such as increasing the number of cases and exploring parameters of FAF images that are more closely related to visual function.

## Data Availability

The datasets used and/or analyzed in the current study are available from the corresponding author on reasonable request.
